# Impact of Laminitis on the Canonical Wnt Signaling Pathway in Basal Epithelial Cells of the Equine Digital Laminae

**DOI:** 10.1371/journal.pone.0056025

**Published:** 2013-02-06

**Authors:** Le Wang, Erica A. Pawlak, Philip J. Johnson, James K. Belknap, Susan Eades, Sharon Stack, Helene Cousin, Samuel J. Black

**Affiliations:** 1 Department of Veterinary and Animal Sciences, University of Massachusetts, Amherst, Massachusetts, United States of America; 2 The University of Missouri College of Veterinary Medicine, Columbia, Missouri, United States of America; 3 The Ohio State University College of Veterinary Medicine, Columbus, Ohio, United States of America; 4 The Louisiana State School of Veterinary Medicine, Baton Rouge, Louisiana, United States of America; 5 The University of Notre Dame, Notre Dame, Indiana, United States of America; University of Liverpool, United Kingdom

## Abstract

The digital laminae is a two layer tissue that attaches the distal phalanx to the inner hoof wall, thus suspending the horse's axial skeleton in the hoof capsule. This tissue fails at the epidermal:dermal junction in laminitic horses, causing crippling disease. Basal epithelial cells line the laminar epidermal:dermal junction, undergo physiological change in laminitic horses, and lose versican gene expression. Versican gene expression is purportedly under control of the canonical Wnt signaling pathway and is a trigger for mesenchymal-to-epithelial transition; thus, its repression in laminar epithelial cells of laminitic horses may be associated with suppression of the canonical Wnt signaling pathway and loss of the epithelial cell phenotype. In support of the former contention, we show, using laminae from healthy horses and horses with carbohydrate overload-induced laminitis, quantitative real-time polymerase chain reaction, Western blotting after sodium dodecylsulfate polyacrylamide gel electrophoresis, and immunofluorescent tissue staining, that positive and negative regulatory components of the canonical Wnt signaling pathway are expressed in laminar basal epithelial cells of healthy horses. Furthermore, expression of positive regulators is suppressed and negative regulators elevated in laminae of laminitic compared to healthy horses. We also show that versican gene expression in the epithelial cells correlates positively with that of β-catenin and T-cell Factor 4, consistent with regulation by the canonical Wnt signaling pathway. In addition, gene and protein expression of β-catenin correlates positively with that of integrin β4 and both are strongly suppressed in laminar basal epithelial cells of laminitic horses, which remain E-cadherin^+^/vimentin^−^, excluding mesenchymal transition as contributing to loss of the adherens junction and hemidesmosome components. We propose that suppression of the canonical Wnt signaling pathway, and accompanying reduced expression of β catenin and integrin β4 in laminar basal epithelial cells reduces cell:cell and cell:basement membrane attachment, thus, destabilizing the laminar epidermal:dermal junction.

## Introduction

The equine digital laminae span between the surface of the distal phalanx and the inner hoof wall, suspending the horse's axial skeleton and attached structures within the hoof capsule. Laminae are composed of 500 to 600 vertical folds of keratinized epidermal tissue, the primary epidermal laminae, which are contiguous with the inner hoof wall, and interdigitated folds of connective tissue, the primary dermal laminae, which are contiguous with the distal phalanx (Fig S1). Each fold of primary epidermal and dermal laminae has 150 to 200 interdigitated folds of secondary laminae resulting in a greatly expanded contact area. The secondary epidermal and dermal laminae join at a basement membrane which is a meshwork of collagen fibers and laminins anchored via hemidesmosomes to basal epithelial cells residing at the boundary of the epidermal laminae [1] and tethered to tensile collagen fibers of the dermal laminae. These are bound by a variety of extracellular matrix components to each other and to integrins expressed by cells on both sides of the membrane thus ensuring integrity of the tissue.

The laminar epidermal:dermal junction is compromised in a variety of disease conditions including septic processes, systemic toxicity, metabolic or endocrinopathic deregulation, traumatic injury and sustained elevated load resulting from contralateral limb injury [2]. These conditions cause the epidermal and dermal laminae to separate to varying degrees, leading to painful and often crippling lameness which can be graded by the Obel scale [3], with Obel grade 1 (OG1) designating lameness that is noticeable at the trot only, OG2 designating lameness noticeable at the walk, OG3 designating lameness that renders the animal reluctant to move and OG4 designating lameness that causes recumbence. Although laminitis has been recognized for some thousands of years [4], mechanisms of pathophysiology leading to the condition have not been resolved.

Failure of the laminar epidermal:dermal junction in horses with starch-gruel induced laminitis, which is a model for natural disease [5], is associated with physiological changes in laminar basal epithelial cells. These include a diminished number of hemidesmosomes per unit area of basement membrane [6], [7], expression of the stress protein calprotectin [8], elevated gene and protein expression of A Disintegrin And Metalloproteinase with Thrombospondin Motifs-4 (ADAMTS-4) [9] and depletion of intracellular versican by a combination of elevated ADAMTS-4 cleavage and suppressed versican gene expression [10]. Versican gene expression is linked to mesenchymal to epithelial transition in cell culture experiments and the proteoglycan induces mesenchymal to epithelial transition of metastatic tumor cells accelerating their proliferation [11]. Consequently, the depletion of versican from laminar basal epithelial cells in laminitic horses may affect epithelial cell lineage commitment and associated functions that are critical to maintaining the laminar epidermal:dermal junction. Furthermore, rat and human versican gene expression can be induced by the β-catenin/TCF transcription complex [12], [13] and thus, is regulated by the canonical Wnt signaling pathway (reviewed in [14]–[16]), raising the possibility that suppressed versican gene expression in laminar basal epithelial cells of horses presenting laminitis may result from perturbations in the canonical Wnt signaling pathway.

The canonical Wnt signaling pathway is one of the fundamental pathways that control cell development, proliferation, differentiation, polarity and motility. The core activity of the pathway is regulation of the production and stability of β-catenin in the cell. This is important because β-catenin mediates canonical Wnt signaling by binding to and activating members of the T cell factor (TCF) transcriptional factor family. The canonical Wnt signaling pathway regulates expression of genes encoding versican [12], [17] and many other proteins. Pathway components that elevate production and stability of β-catenin are called “positive regulators”. These are: (i) Wnt, named after Wingless in Drosophila and integration 1 in mouse breast tumors, which is a glycoprotein that activates the pathway, (ii) its receptor, frizzled (FZD), (iii) its co-receptor, low density lipoprotein receptor related protein (LRP); (iv) disheveled (Dsh) which together with the Wnt/FZD/LRP complex forms a signalosome that inhibits glycogen synthase kinase 3β (GSK3β), thus stabilizing β-catenin by preventing its phosphorylation and therefore its ubiquitination and proteasomal degradation, (v) protein phosphatase 1 (PP1) and protein kinase B (Akt), which also prevent β-catenin phosphorylation, and (vi) TCF which in complex with transcriptional co-activator β-catenin induces target gene expression. Pathway components that diminish expression of β-catenin and elevate its degradation are called “negative regulators”. These are: (i) Dickkopf (DKK), which antagonizes Wnt signaling by binding the co-receptor LRP, (ii) components of the β-catenin degradation complex, namely, the scaffolding proteins Axin and *adenomatous polyposis coli* gene product (APC) which bind β-catenin, and (iii) casein kinase 1α (CK1α) and GSK3β which jointly phosphorylate bound β-catenin leading to its subsequent ubiquitination and proteasomal degradation.

Here we determine: i) whether suppressed versican gene and protein expression which occurs in LBEC of laminitic horses [10] is accompanied by changes in expression of E-cadherin and vimentin, and hence with a change in the lineage commitment of the cells, ii) whether positive and negative regulatory components of the canonical Wnt signaling pathway are expressed in LBEC and if so, how expression levels change during the development of laminitis, and iii) whether the development of laminitis is accompanied by changes in expression of components of adherens junctions (E-cadherin and β-catenin) and hemidesmosomes (integrin α6 and integrin β4).

## Materials and Methods

### Ethics Statement

This study was carried out in strict accordance with the recommendations in the Guide for the Care and Use of Laboratory Animals of the National Institutes of Health. The Protocol was approved by the Animal Care and Use Committee of the University of Missouri, College of Veterinary Medicine, Protocol # 4329 and of the Louisiana State School of Veterinary Medicine, Protocol # 11056.

### Animal and tissue collection

Healthy horses (3–12 years of age; 341–524 kg) with no previous history or signs of laminitis were used. Prior to inducing experimental laminitis all horses were subjected to a complete physical examination (including rectal temperature, heart rate, respiratory rate, auscultation of abdominal sounds, digital pulses and evaluation with hoof testers) and gait evaluation (horses were walked and trotted in straight lines and walked in circles in each direction). At the University of Missouri College of Veterinary Medicine, horses were administered carbohydrate gruel (85% cornstarch and 15% wood flour [17.6 g/kg body weight]) by nasogastric tube, or similarly administered 6 liters of deionized water as previously described [5], [18]. General anesthesia [19] was induced in starch gruel treated horses at the onset of OG1-lameness (shifting weight between feet incessantly and demonstrating a short, bilaterally stilted gait at trot) (n = 6;4 females, 2 gelded males) or upon development of OG3-lameness (reluctance to walk) (n = 5; 3 females, 2 gelded males) and, in the case of control horses (n = 8; 4 females and 4 gelded males), 24 hours after administration of water. In all cases, front hoof laminae were dissected from excised hoofs as previously described [19], [20]. Samples of laminae were flash frozen in liquid nitrogen for molecular and biochemical analyses. For immunofluorescence microscopy, segments were embedded in O.C.T. (Sakura Finetek USA, Inc., Torrance, CA) and frozen over dry ice. At the Louisiana State School of Veterinary Medicine an additional 3 healthy horses were subjected to the same laminitis-inducing protocol, and upon presenting OG3 lameness, segments of front hoof dorsal laminae were harvested and similarly frozen in O.C.T. for immunofluorescence microscopy.

### RNA extraction

RNA was isolated from three separate sections of front hoof dorsal laminae from each horse using Stratagene Absolutely RNA kit (Stratagene, La Jolla, CA) according to manufacturer's instructions. Briefly, flash frozen tissue was pulverized in a dry ice-chilled slammer (Biospec Products, Inc., Bartlesville, OK) and homogenized in the lysis buffer provided in the kit, then processed accordingly. The purity and concentration of RNA were determined by NanoDrop 1000 (Thermo Scientific, Wilmington, DE) and consistently yielded ratios of A260:A280 and A260:A230 close to 2.0. Integrity of isolated RNA was confirmed by electrophoresis on a 1.0% agarose gel and staining with SYBRSafe DNA Gel Stain (Molecular Probes, Eugene, OR).

### Real-time quantitative-PCR

cDNA was synthesized using Quanta qScript cDNA Synthesis Kit (Quanta BioSciences, Gaithersburg, MD). Primer sets are listed in Table 1. RT-qPCR reactions were performed using SYBR Premix Ex Taq (Applied Biosystems, Foster City, CA) according to manufacturer's instructions and data were recorded by Stratagene MX 3005p (Stratagene, La Jolla, CA). Each sample was run in duplicate and their Ct values were analyzed using the ΔΔCT method of analysis [21]. We determined the expression patterns of glyceraldehyde 3-phosphate dehydrogenase (GapDH), β actin, β2 microglobulin and TATA Box binding protein in laminar samples from control horses and horses presenting OG1 and OG3 lameness after administration of starch gruel. Expression of these genes did not differ significantly between groups. However, expression patterns of β2 microglobulin and GapDH had the lowest variation within groups and we normalized gene expression to GapDH as previously reported [9]. Contamination and formation of primer dimers were monitored by a dissociation curve. All primer pairs were confirmed to amplify a single band by running products in 1.2% agarose gel and staining with SYBRSafe DNA Gel Stain (Molecular Probes, Eugene, OR) and validating products by sequencing (Genewiz, Inc. South Plainfield, NJ).

**Table 1:** Primer sequences utilized in RT-qPCR evaluation of gene expression.

Domain Name	Sequence (F-forward 5′; R–reverse 5′)	Amplicon length (bp)	Primer efficiency	R^2^
Akt2	F-AGGACCCCATGGACTACAAGT-3′ R-CCAGGAGTTTGAGATAGTCGAA-3′	126	95%	0.99
APC	F- GACCAGAAGGCAATTGGAATA-3′ R- GGACTGCAAAAGCTGTCGTAT-3′	160	99%	0.98
Axin1	F-GATCTTCCGGGACAAAGAAG-3′ R-GCATGGTAGGGTCTTGAATGA-3′	147	99%	0.98
β-catenin	F-CCCTGAACTGACAAAACTGCTA-3′ R-AATAGCAGACACCATCTGAGGA-3′	136	98%	0.99
CKIα	F-TTGGGCGTCACTGTAATAAGTT-3′ R-GCAGTGCCAGTGAGATTTTTAT-3′	118	96%	0.99
Dsh	F-ACAACGAGACCAGCACAGAGT-3′ R-GTGTTGTCATCCTCGTCTGAGT-3′	205	101%	0.99
E-cadherin	F-CATTGTGTACTCCATCCTCAGC-3′ R-TGTACTTAAGCCCTCACCTTGA-3′	175	99%	0.99
FZD4	F-CTGCTGTTGCTGTTGCTACTC-3′ R-GATGAGAGGCGTGAAAGTTGT-3′	189	96%	0.98
GSK3β	F-ATCTGCCATCGGGATATTAAAC-3′ R-ATACGAAACATTCGGTTCTCCT-3′	120	102%	0.97
Integrin α6	F-AGTCTGCACATCTTCTTCCTGA-3′ R-CGCTCCAATCACTATATCTTGC-3′	122	100%	0.99
Integrin β4	F-GATGTGAATGAGTTCCGGAGT-3′ R-GTCAGCCTCGTAGTGGAAGG-3′	183	100%	0.99
LRP6	F-GCCAAATGGACTAACTTTGGAT-3′ R-GTGTCCTCAAATAACGTCAAGG-3′	159	102%	0.99
PP1	F-AAAACCTTCACCGACTGCTT-3′ R-ATACGCCGAATCTGTTCCAT-3′	119	97%	0.99
TCF4	F-TTCTTTCAGCCAACAGACATTC-3′ R-AGTTGCAGACTGGACAGGAAG-3	128	101%	0.99
Versican	F-CCTGCAATTACCATCTCACCTA-3′ R -CAGGGAGTTGATTTCATAACGA-3′	122	92.1%	0.99
Vimentin	F-ACGTTCGTCAGCAGTATGAAA-3′ R-GTTAGCAGCCTCAGAGAGGTC-3′	98	97%	0.99
Wnt4	F-AGCTGGAAAAGTGTGGCTGT-3′ R-GTCCACAAACGACTGTGAGAAG-3′	119	103%	0.97

### Protein extraction

0.35 g snap frozen tissue was pulverized in a dry ice-chilled slammer (Biospec Products, Inc., Bartlesville, OK) immediately homogenized in 10 ml of extraction buffer (50 mM Tris pH7.0, 150 mM NaCl, 5 mM ethylenediaminetetraacetic acid, 0.5% NP-40 containing 10 µM E64, 1.5 µM pepstatin A and 1 mM phenylmethanesulfonyl fluoride) on ice, extracted overnight at 4°C and protein concentration determined by Bradford Assay (Bio Rad Life Sciences, Hercules, CA).

### Sodium dodecylsulfate polyacrylamide electrophoresis (SDS-PAGE) and Western Blotting

An aliquot (30 µg protein content) of laminar extract from each horse was boiled in reducing (5 mM 2-mercaptoethanol) Laemmli sample buffer (Bio Rad Life Sciences, Hercules, CA) for 5 min and subjected to SDS-PAGE in a 4% (w/v) polyacrylamide stacking gel with a 10% (w/v) polyacrylamide gel as previously described [22]. Proteins were transferred to polyvinylidene fluoride membrane (Millipore, Billerica, MA) by electroblotting. The membrane was blocked with 5% dry milk in PBS with 0.05% tween-20 for 1 hr, washed with PBS with 0.1% tween-20 for 30 min and then incubated with appropriate primary antibodies (Table 2) overnight at 4°C to detect integrin β4, β-catenin, GSK3β, phosphorylation of GSK3β S9, E-cadherin, and β actin, which was used as a loading control. Bound primary antibody was revealed with appropriate secondary antibodies (Table 2). Detection was performed using enhanced chemiluminescence (Bio Rad Life Sciences, Hercules, CA) and visualized with G: Box (Syngene, Frederick, MD) with Gene Tools (Syngene, Frederick, MD) for quantification.

**Table 2:** Antibodies used for Western blot analysis of protein expression.

Primary Antibody	Host Species and Epitope	Dilution	Company
anti-β actin	Mouse monoclonal to beta Actin-Loading Control	1∶20000	AbCam ab8226
Anti-E-cadherin	Mouse monoclonal[M168] to C terminal region of mouse E cadherin	1∶1000	AbCam ab76055
Anti-β-catenin	Rabbit polyclonal to aa 768–781 of human or mouse β-catenin	1∶4000	AbCam ab6302
anti-GSK3β	Rabbit polyclonal to aa335–349 of rat GSK3β	1∶500	Millipore # 07-1413
anti-Integrin β4	Mouse IgG1 to human integrin β4 aa. 1612–1821	1∶500	BD #611232
anti-serine-9-phospho-GSK3β (S9)	Rabbit monoclonal[ EPR2286Y] antibody to human GSK3β	1∶5000	Millipore # 04-1075
anti-Vimentin	Mouse monoclonal [V9]	1∶1000	AbCam ab8069
**Secondary Antibody**			
anti-Mouse Ig	Sheep polyclonal to mouse IgG-H&L (HRP)	1∶10000	AbCam ab6808
anti-Rabbit Ig	ECL Rabbit IgG, HRP-Linked Whole Ab (from donkey)	1∶4000	Jackson ImmunoResearch #711-515-152

### Immunofluorescence localization

10 µm frozen sections were cut from Tissue-Tek O.C.T-embedded tissues and affixed to Fisher Superfrost Plus glass slides (Fisher Scientific, Fair Lawn, NJ). Indirect immunofluorescence staining was performed using a combination of antibodies that separately stain integrin β4, laminin, β-catenin, E-cadherin, Wnt4, FZD4, and vimentin (Table 3) together with DAPI, a fluorescent DNA-intercalating agent. Briefly, slides were blocked with 5% BSA in PBS with 0.001% tween-20 and treated with optimal dilutions of primary antibodies, described in Table 3, for one hour at room temperature. Sections were washed and treated for one hour at room temperature with secondary antibodies (Table 3) along with a 1∶2000 dilution of DAPI. All slides were imaged using a Carl Zeiss inverted microscope with apotome grid, ultraviolet, blue and green excitation light and 20x or 63x objectives Zeiss MOT200 with Zeiss Apotome (MicroImaging, Inc., Thornwood, NY).

**Table 3:** Antibodies used for Immunofluorescent Localization.

Primary Antibody	Host Species & Epitope	Dilution	Company
anti-β-catenin	Rabbit polyclonal to aa 768–781 of human or mouse β-catenin	1∶100	AbCam ab6302
anti-E-cadherin	Mouse monoclonal[M168] to C terminal region of mouse E-cadherin	1∶100	AbCam ab76055
anti-FZD4	Rabbit polyclonal to 488–537 of human Frizzled 4	1∶100	AbCam ab83024
anti-Integrin β4	Mouse IgG1 to human integrin β4 aa. 1612–1821	1∶50	BD #611232
anti-Laminin	Rabbit polyclonal to laminin	1∶100	AbCam ab11575
anti-Wnt4	Rabbit polyclonal to the center region of human Wnt4	1∶50	AbCam ab91226
anti-Vimentin	Rabbit polyclonal to C-terminus of human vimentin	1∶100	AbCam ab45939
**Secondary Antibody**			
anti-Mouse Ig	Donkey polyclonal to mouse IgG H&L – conjugated with a fluorochrome with EX/EM* of 591/616 nm	1∶200	Jackson ImmunoResearch #715-515-150
anti-Mouse Ig	Donkey polyclonal to mouse IgG H&L – conjugated with a fluorochrome with EX/EM* of 493/518 nm	1∶200	Jackson ImmunoResearch #715-485-150
anti-Rabbit Ig	Donkey polyclonal to rabbit IgG H&L – conjugated with a flurochrome with Ex/EM* of 591/616 nm	1∶200	Jackson ImmunoResearch #711-515-152
anti-Rabbit Ig	Donkey polyclonal to rabbit IgG H&L – conjugated with a fluorochrome with EX/EM* of 493/518 nm	1∶200	Jackson ImmunoResearch #711-485-152

### Statistical analysis

Statistical analyses of gene expression and Western blot data were performed using Graph Pad Prism Version 5 (Graph Pad Software, San Diego, CA). All gene expression data were normally distributed as determined by Column Statistics using the D'Agostino and Pearson Omnibus Normality Test. Protein expression data of integrin β4, β-catenin, vimentin, E-cadherin and serine-9-phospho-GSK3β, were also normally distributed. GSK3β protein expression data were not normally distributed. Normally distributed data were analyzed by one-way ANOVA with Dunnett's post hoc test, and by Pearson product moment correlation co-efficient analyses with two-tailed 95% confidence. Data that were not normally distributed were analyzed using the Kruskal–Wallis one-way ANOVA with Dunn's multiple comparisons test. In all tests a P value of <0.05 was considered significant.

## Results

### Integrin β4, β-catenin and vimentin gene and protein expression in healthy and laminitic laminae

**Figure 1:**
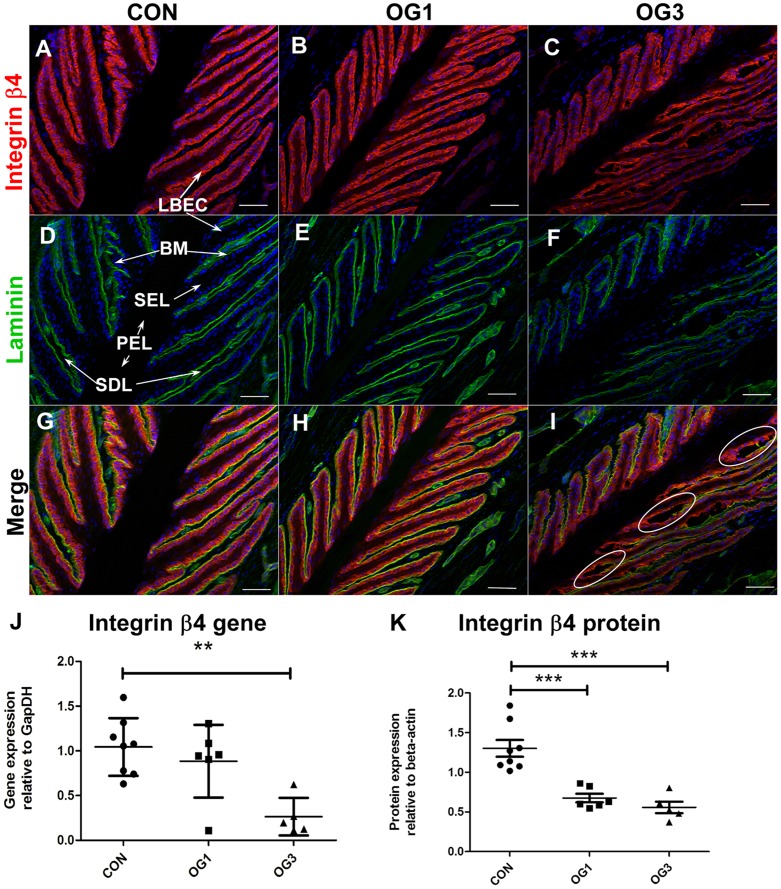
Distribution and expression of integrin β4 in digital laminae of healthy and laminitic horses. **A–I** - 10 µm sections of frozen laminae from: **A, D, G** - a representative (n = 3) healthy horse (CON) (**D** – LBEC = laminar basal epithelial cell, BM = basement membrane, SEL = secondary epidermal laminae, PEL = primary epidermal laminae, SDL = secondary dermal laminae); **B, E, H** - a representative (n = 4) horse with OG1-lameness (OG1); and **C, F, I** - a representative (n = 3) horse with OG3-lameness (OG3) were stained with antibodies against integrin β4 (A,B,C - red) and against laminin (D,E,F green). Red and Green fluorescence merge in yellow (G, H, I). Nuclei are stained blue with DAPI. White circles indicate areas of dissociation between basal epithelial cells and basement membrane laminin. Scale bars 50 µm. **J** - Quantitation of integrin β4 and gene expression relative to the gene expression of GapDH. **K** - Quantitation of integrin β4 protein expression relative to β actin. Horizontal lines indicate mean ± standard error of mean of n = 8 CON, n = 6 OG1 and n = 5 OG3, ** = p<0.01, ^***^ = p<0.001 as calculated by one-way ANOVA.

In the laminae of both healthy horses and of horses with starch gruel-induced laminitis, expression of the hemidesmosome component integrin β4 is restricted to the single layer of basal epithelial cells of the secondary epidermal laminae (Fig. 1A, B and C; stained red with specific antibody). The protein is detected throughout the basal epithelial cell cytoplasm, and a portion is present at the cell margin adjacent to the basement membrane (Fig. 1D, E and F; stained green with anti-pan-laminin), as indicated by co-localization (Fig. 1G, H and I; stained yellow as a result of merged green and red stains at the integrin β4:laminin interface). The level of expression of the gene encoding integrin β4 is significantly reduced in laminae of horses presenting OG3 lameness compared to that in healthy horses (Fig. 1J). In addition, there is a significant drop in integrin β4 protein concentration in extracts of laminae from horses presenting OG1 and OG3 lameness compared to those prepared from healthy horses (Fig. 1K; Western blots shown in Fig S2, panel A). In horses with starch-gruel induced laminitis, suppressed integrin β4 gene and protein expression (Fig. 1J and K) is accompanied by separation of the basement membrane and basal epithelial cells in focal areas proximal to the primary epidermal laminae (Fig. 1I; indicated by white ovals) a condition that is not observed in sections of laminae from healthy horses or horses presenting OG1 lameness.

**Figure 2:**
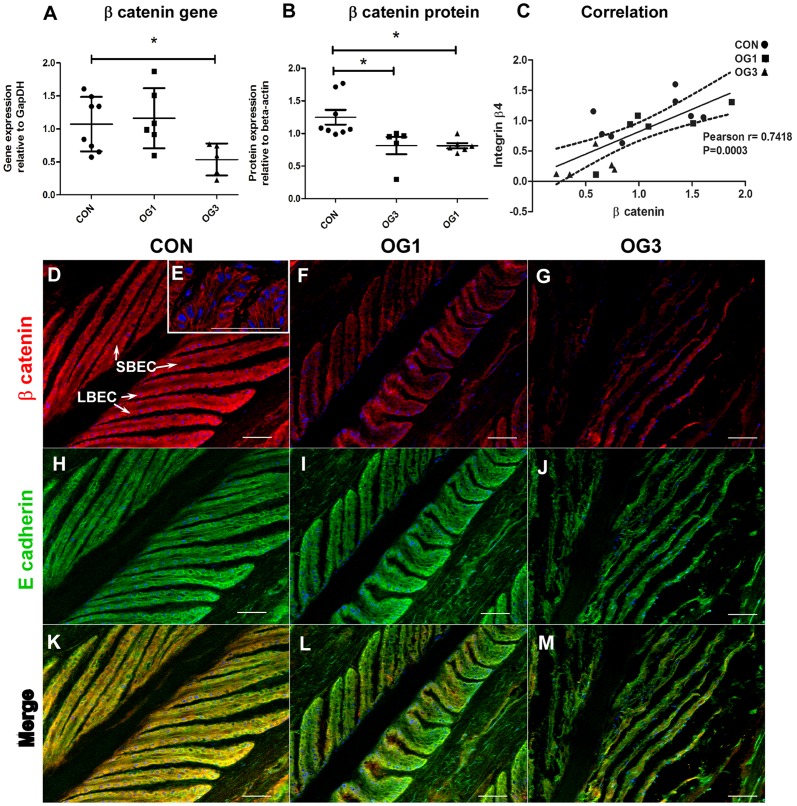
Distribution and expression of β-catenin and E-cadherin in digital laminae of healthy and laminitic horses. **A** - Quantitation of β-catenin gene expression relative to the gene expression of GapDH. **B** - Quantitation of β-catenin protein expression relative to the protein expression of β actin. Horizontal lines indicate mean ± standard error of mean * = p<0.05 as calculated by one-way ANOVA. **C** - Correlation between expression of genes encoding β-catenin and integrin β4: Pearson r = 0.7418, p = 0.0003. Solid line represents the predicted positive correlation by linear regression; Area within the dotted lines represents 95% confidence. n = 8 CON, n = 6 OG1, and n = 5 OG3. **D-M** (20X objective) - 10 µm sections of frozen laminae from: **D, H, K** - a representative (n = 3) healthy horse (CON) (**D** – LBEC = laminar basal epithelial cell, SBEC = supra-basal epithelial cell) ; **F, I, L** - a representative (n = 4) horse with OG1-lameness (OG1) and **G, J, M** - a representative (n = 3) horse with OG3-lameness (OG3) were stained with antibodies against β-catenin (D, F, G- red) and E-cadherin (H, I, J - green). Red and Green fluorescence merge in yellow (K, L, M). Nuclei are stained blue with DAPI. Scale bars 50 µm. **E** (63X objective) – β-catenin stained in red, nuclei stained blue.

Reduced expression of integrin β4 in laminitic laminae is accompanied by reduced gene and protein expression of the adherens junction and canonical Wnt signaling pathway component β-catenin (Fig. 2A and 2B; Western blots shown in Fig S2, panel B). In addition, the level of expression of the gene encoding β-catenin in laminae correlates strongly and positively with that of the gene encoding integrin β4 (Fig. 2C Pearson's r = 0.74; p = 0.0003) consistent with the possibility that expression of these genes is co-regulated. Like integrin β4, β-catenin is highly expressed in laminar basal epithelial cells of healthy and laminitic horses (Fig. 2 D–G; stained red with specific antibody), where it is richly present around cell margins (Fig. 2E). E-cadherin is also highly expressed in these cells (Fig. 2H–J; stained green with specific antibody) and for the most part co-localizes with β-catenin (Fig. 2K–M; stained yellow as a result of merged green and red stains) to which it is tethered in adherens junctions.

**Figure 3:**
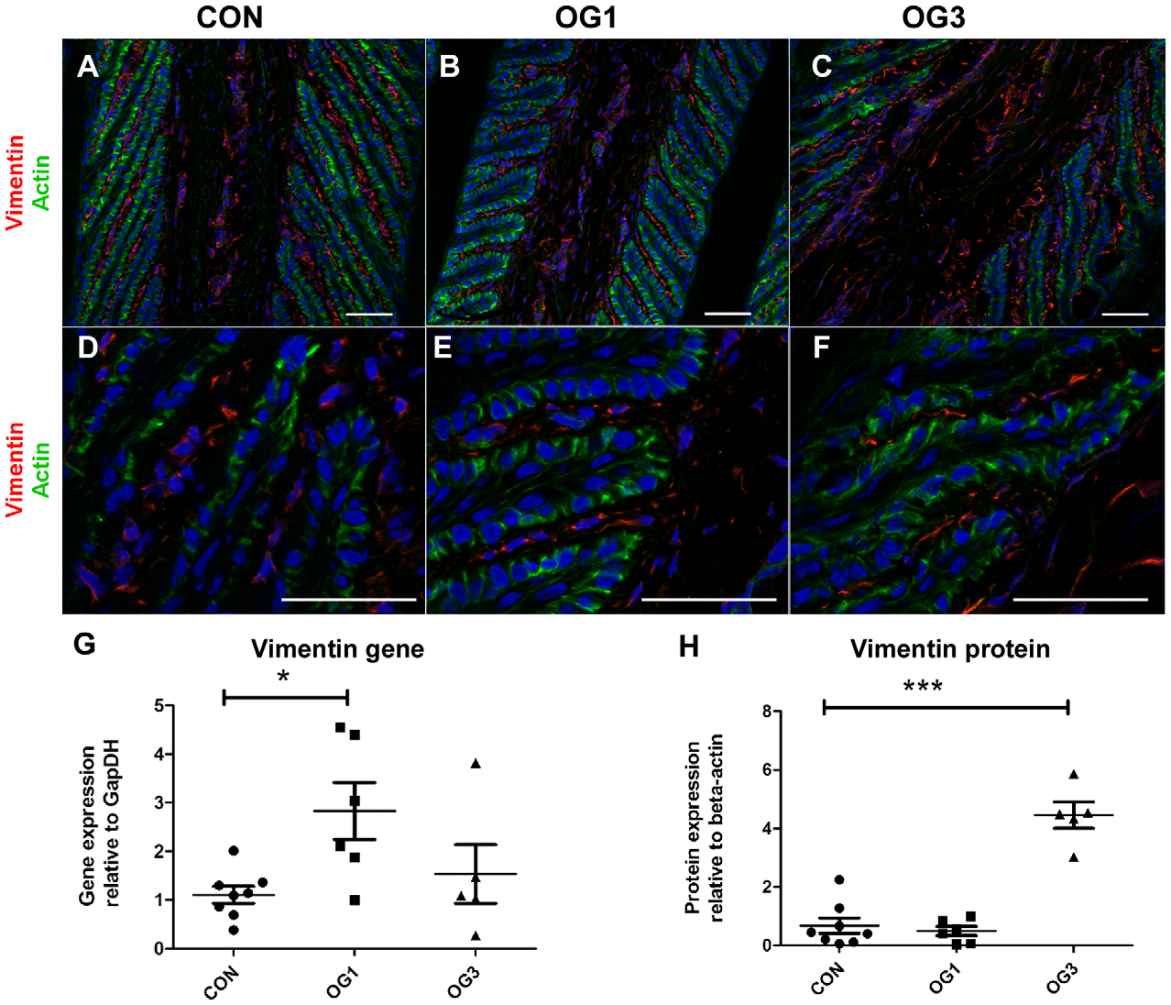
Distribution and expression of vimentin in digital laminae of healthy and laminitic horses. **A, B, C** (20X objective), **D, E, F** (63X objective) - 10 µm sections of frozen laminae from a representative (n = 3) healthy horse (A,D), a representative (n = 4) horse with OG1-lameness (B,E) and a representative (n = 3) horse with OG3-lameness (C,F) were stained with antibodies against vimentin (red) and with phalloidin-FITC (green). Nuclei are stained blue with DAPI. Scale bars 50 µm. **G** - Quantitation of vimentin gene expression relative to the gene expression of GapDH. **H** - Quantitation of vimentin protein expression relative to the protein expression of β actin. Horizontal lines indicate mean ± standard error of mean * = p<0.05 as calculated by one-way ANOVA. n = 8 CON, n = 6 OG1, and n = 5 OG3.

Gene (at OG1) and protein (at OG3) expression of the type III intermediate filament protein vimentin, which is a marker of mesenchymal cells [23], is significantly elevated in the laminae of laminitic horses compared to laminae of healthy horses (Figs 3G and 3H; Western blots shown in Fig S2, panel C). Vimentin protein (stained red with specific antibody) is expressed by cells of the primary and secondary dermal laminae of healthy and laminitic horses but not by cells of the epidermal laminae (Figs 3A–F). Laminar basal epithelial cells from healthy horses (Fig. 3A and D) and from horses presenting OG1 (Fig. 3B and E) or OG3 (Fig. 3C and F) lameness have a well-developed cortical actin cytoskeleton which stains green with phalloidin-FITC and do not express vimentin. Hence, elevated vimentin gene and protein expression in laminae of laminitic horses (Figs 3G and 3H) is restricted to cells of the dermal connective tissue.

### Expression of canonical Wnt signaling pathway components in healthy and laminitic laminae

**Figure 4:**
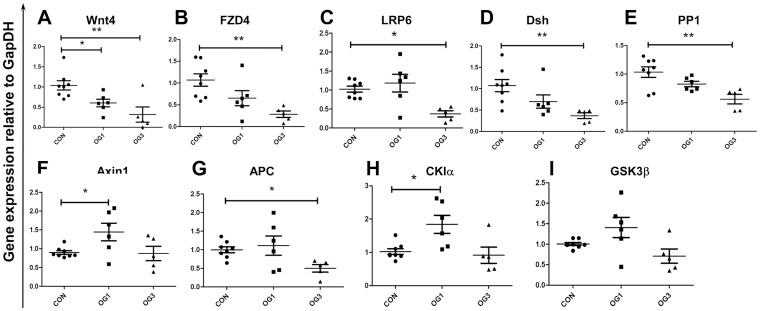
Gene expression of Wnt signaling pathway regulators: Expression of genes encoding **A** - Wnt4, **B** - FZD4, **C** - LRP6, **D** - Dsh, **E** - PP1, **F** - Axin1, **G** - APC, **H** - CKIα, **I** - GSK3β relative to the gene expression of GapDH. Horizontal lines indicate mean ± standard error of mean of n = 8 CON, n = 6 OG1, and n = 5 OG3, animals. * = p<0.05, ** = p<0.01, as calculated by one-way ANOVA.

Levels of β-catenin protein expression are regulated by the canonical Wnt signaling pathway. Levels of β-catenin gene expression may also be regulated, directly and indirectly, by the canonical Wnt signaling pathway [24]. Consistent with suppressed β-catenin gene and protein expression in laminitic laminae, expression of genes encoding positive canonical Wnt signaling pathway regulators, namely Wnt4, FZD4, LRP6, Dsh and PP1, is also suppressed (Fig. 4A–E). Furthermore, immunofluorescence staining shows that Wnt4 is mainly expressed in the basal and supra-basal epithelial cells of the secondary epidermal lamellae of healthy and laminitic horses (Fig S3A, B, C; stained red with specific antibody), while FZD4 is mainly expressed in laminar basal epithelial cells (Fig S3D, E, F; stained red with specific antibody).

**Figure 5:**
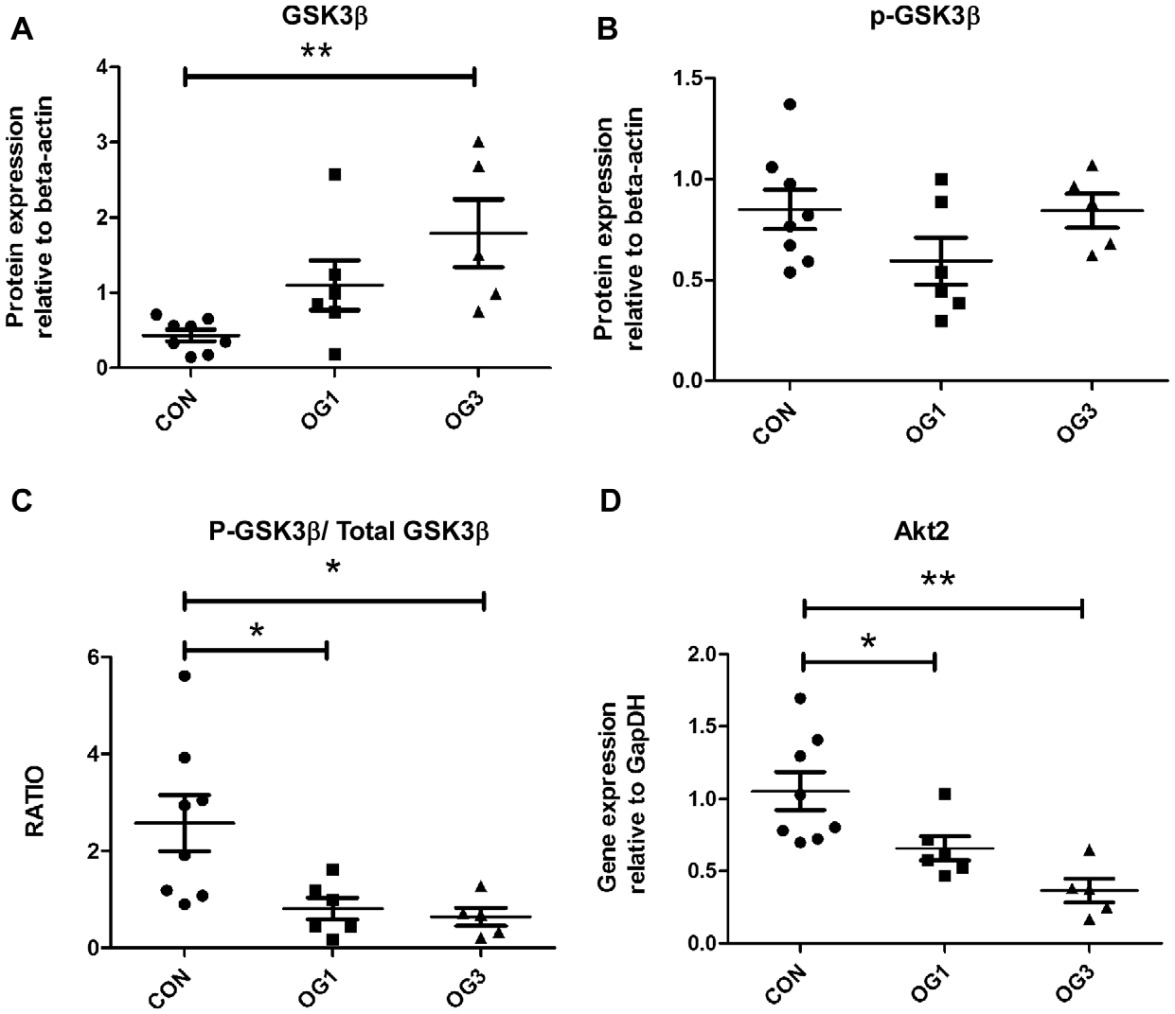
Protein expression and phosphorylation of GSK3β: **A** Total GSK3βprotein expression relative to β actin; **B** – Phosphorylated S9 GSK3βprotein (p-GSK3β) expression relative to β actin; **C** -Ratio of p-GSK3β to total GSK3β; **D** - Akt2 gene expression relative to GapDH. Horizontal lines indicate mean ± standard error of mean of n = 8 CON, n = 6 OG1, and n = 5 OG3 animals. * = p<0.05, ** = p<0.01as calculated by one-way ANOVA.

Whereas expression of genes encoding positive regulators of the canonical Wnt signaling pathway is suppressed in the laminae of laminitic horses (Fig. 4A–E), expression of genes encoding components of the β-catenin degradation complex (negative regulators of the canonical Wnt signaling pathway) is either transitorily elevated, namely, Axin 1 and CK1α (Fig. 4F and 4H), suppressed, namely, APC (Fig. 4G), or unaltered, namely, GSK3β (Fig. 4I). Although the level of GSK3β gene expression is unaltered in the laminae of laminitic horses relative to that in the laminae of healthy horses, the level of GSK3β protein increases in laminitic laminae (Fig. 5A), that of serine-9-phospho-GSK3β (p-GSK3β) is unchanged (Fig. 5B), and ratio of p-GSK3β to total GSK3β decreases (Fig. 5C; Western blots shown in Fig S2, panel D and E). Expression of the gene encoding Akt2, which is known to phosphorylate GSK3β and accelerate its degradation [25], was also reduced in laminae of laminitic compared to healthy horses (Fig. 5D).

**Figure 6:**
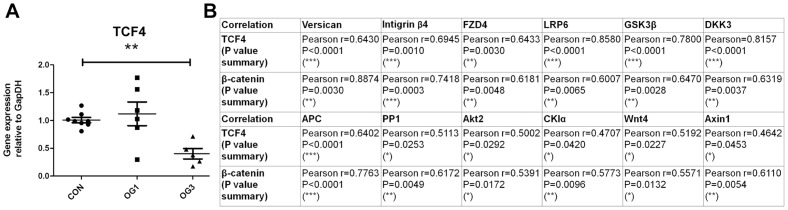
TCF4 gene expression and correlation summary: **A** - Gene expression of TCF4 relative to the gene expression of GapDH. Horizontal lines indicate mean ± standard error of mean of n = 8 CON, n = 6 OG1, and n = 5 OG3 animals. **B** - Summary of correlation between expression of indicated genes. ^*^ = p<0.05, ** = p<0.01, ^***^ = p<0.001 as calculated by one-way ANOVA.

Finally, expression of the gene encoding TCF4, which in complex with transcriptional co-activator β-catenin induces target gene expression in the canonical Wnt signaling pathway [26], is suppressed in laminitic laminae (Fig. 6A). Furthermore, comparative analyses of gene expression in laminae of all control, OG1 and OG3 animals show there are strong positive correlations between the expression of genes encoding β-catenin and TCF4 and those encoding some positive and negative regulators of the canonical Wnt signaling pathway (Fig. 6B) consistent with feed forward regulatory loops. Most notable among these are TCF4 and the positive pathway regulator LRP-6 (Pearson's r = 0.8580, P<0.0001), and TCF4 and the negative regulators GSK3β (Pearson's r = 0.7800, P<0.0001) and APC (Pearson's r = 0.8174, P<0.0001).

A strong positive correlation was also observed between the gene encoding TCF4 and that encoding versican (Pearson's r = 0.89, P<0.0001), and a moderate positive correlation between the gene encoding β-catenin and that encoding versican (Pearson's r = 0.64, P = 0.003). In addition, there was a strong positive correlation between expression of the gene encoding TCF4 and that encoding integrin β4 (Pearson's r = 0.69, P = 0.001), and between the gene encoding and β-catenin and integrin β4 (Pearson's r = 0.74, P = 0.0003).

## Discussion

Studies reported above show that genes and proteins encoding multiple positive regulators of the Canonical Wnt Signaling Pathway were decreased in the laminae of horses affected by laminitis. In addition, many of the known negative regulators of the wnt pathway were increased in the same conditions, suggesting that the wnt signaling pathway is negatively regulated during the progression of laminitis. This possibility is further supported by evidence that versican, one of the known targets of the Wnt pathway [12], [13], is significantly decreased during laminitis progression [10]. In addition, expression of versican in laminae correlated positively with expression of genes encoding β-catenin (Pearson's r = 0.64) and TCF4 (Pearson's r = 0.89, p<0.0001) which jointly constitute the co-transcriptional activator that regulates gene expression in the canonical Wnt signaling pathway. Determination of the importance of wnt signaling in the progression of laminitis will require additional studies that could involve the use of activators of the pathway, for example GSK3 inhibitors, to see if those can delay or prevent the progression of the disease as discussed below.

Versican gene and protein expression is associated with mesenchymal-to-epithelial cell transition in transfected NIH 3T3 cells [27], [28], and versican in extracellular matrix has been demonstrated to promote mesenchymal-to-epithelial transition of metastatic tumor cells [29]. In addition, during development of the vertebrate heart tube, ADAMTS-mediated cleavage of versican is associated with the formation of endocardial cushion mesenchyme [30]. However, in spite of versican depletion from laminar basal epithelial cells of laminitic horses [10], the cells did not lose their epithelial phenotype or acquire characteristics of mesenchymal cells evidenced by the sustained expression of the epithelial cell marker E-cadherin and lack of expression of the mesenchymal cell marker vimentin. This is perhaps not surprising because epithelial-to-mesenchymal transition is dependent on the canonical Wnt signaling pathway [31], which the data presented here suggest is suppressed in laminar basal epithelial cells of laminitic horses.

Although vimentin gene and protein were not expressed in laminar basal epithelial cells of healthy or laminitic horses, vimentin was strongly expressed by connective tissue cells in the dermal laminae and expression was elevated in laminitic horses. Vimentin is a type III intermediate filament protein present in mesenchymal cells. In addition to expression by cells undergoing epithelial to mesenchymal transition, it is constitutively expressed by resting fibroblasts and expression is elevated in stimulated [32] and stressed cells [33]. Its elevated expression in laminar dermal cells of laminitic horses is consistent with the previously shown inflammatory response occurring in this tissue at the onset of lameness [8], [19], which may elicit a stress/repair response in dermal fibroblasts.

Despite the absence of epithelial-to-mesenchymal transition in the laminar basal epithelial cells of laminitic horses, there was evidence of impaired function. The diminished presence of positive regulators and elevated presence of negative regulators of the canonical Wnt signaling pathway in the laminar basal epithelial cells of laminitic horses correlated with diminished expression of β-catenin and integrin β4, which are components of adherens junctions and hemidesmosomes respectively [34], [35] and are thus required for basal epithelial cell:cell and cell:basement membrane attachment. Based on these observations, we propose that suppression of the canonical Wnt signaling pathway in the laminar basal epithelial cells and accompanying reduced integrin β4 gene and protein expression, is a contributing factor to the reduction in number of hemidesmosomes per unit area of epithelial cell/basement membrane interface reported in laminitis [7].

Although the expression of genes encoding β catenin and integrin β4 was strongly positively correlated in equine laminae (Pearson's r = 0.74, p = 0.0003), direct regulation of integrin β4 gene expression by the canonical Wnt signaling pathway was not demonstrated, and there is no report of this in the literature. In contrast, cross talk has been reported between the canonical Wnt signaling pathway and other signaling pathways [36], [37] and multiple lines of evidence exist that the Wnt pathway is regulated by integrins. For example, beta1 integrin can activate Wnt via the integrin linked kinase (ILK) [38] and the adaptor protein growth factor receptor-bound-2 (Grb-2) has been shown to be recruited by integrin β4 as a result of its interaction with collagen in the extracellular matrix [39] and to enhance β catenin-dependent Wnt signaling [40]. Additional studies are required to establish whether diminished integrin β4-dependent signaling causes down regulation of the canonical Wnt signaling pathway in laminae of laminitic horses, or vice versa, or whether both pathways are down regulated by laminitis-associated changes in other signal pathways.

Suppressed canonical Wnt signaling may not solely be a feature of starch gruel-induced laminitis. In support of this possibility, it has been shown that the number of hemidesmosomes per micrometer of basement membrane is decreased in ponies with insulin-induced laminitis [41], as it is in horses with oligofructose-induced laminitis [7]. In addition, preliminary proteomic studies show that the level of β-catenin is lower in extracts of laminae from horses with hyperinsulinemia-induced laminitis compared to extracts of laminae from healthy horses (Dr. Hannah Galantino-Homer, Personal Communication). It is important to point out that the diminished presence of β-catenin in extracts of laminae from hyperinsulinemic animals does not necessarily mean that the canonical Wnt signaling pathway is suppressed in the laminae of these animals; given that β-catenin is sequestered by E-cadherin [42], a reduction in β-catenin expression could be compensated by a matched reduction in E-cadherin expression. Thus, a detailed analysis of canonical Wnt signaling pathway components and regulators is warranted in laminae of horses presenting laminitis of diverse etiology. It is worthy of note that cross talk between insulin/insulin like growth factor receptor signaling and the Wnt signaling pathway has been established [43] although non-canonical Wnt signaling rather than the canonical pathway is linked to insulin resistance [44], [45].

If suppression of the canonical Wnt signaling pathway in laminar basal epithelial cells is a feature of laminitis of diverse etiology, pathway agonists may help to ameliorate laminar pathology. In this regard, expression of genes encoding the adherens junction component E-cadherin and the hemidesmosome component integrin α6 was not suppressed in horses with starch gruel-induced laminitis (Fig S4) and E-cadherin protein expression was not suppressed (Fig S2, panel F) supporting the possibility that adherens junctions and hemidesmosomes might be restored by sustaining β-catenin expression, e.g., through administration of agents that inhibit GSK3β [46]. This attractive possibility is contradicted by studies showing that protein expression of integrin α6 and anchoring filaments BP180 and laminin 5 is reduced in horses with oligofructose-induced laminitis based on immunofluorescence staining [47], consistent with complex disruption of the hemidesmosome. However, it is possible that the reduced expression of integrin β4 affects expression of other hemidesmosome components. Indeed, murine keratinocytes that lack integrin β4 also lack other hemidesmosome components [48]. Clearly if suppression of the canonical Wnt signaling pathway proves to be a feature of laminitis irrespective of etiology, it will be important to determine which, and to what extent, hemidesmosome, adherens junction and desmosome components are affected in laminar basal epithelial cells of the laminitic animals and to determine the impact of sustaining β-catenin expression on the attachment of the basal epithelial cells to each other and to the basement membrane. Similarly, it will be important to identify the physiological process that results in diminished expression of β-catenin in laminae of laminitic horses.

## Supporting Information

Figure S1 **The anatomy of the hoof wall (Design: Chris Pollitt, Art. John McDougall):** The Figure is reproduced by permission of Dr. C. Pollitt. Sections of laminae presented in the paper are from the mid dorsal front hoof laminae and the region is delineated by the oval shown in this figure.
(TIF)

Figure S2 **Protein expression in NP-40 extracts of healthy and laminitic equine digital laminae:** Western blots of 0.5% NP-40 extracts (30 ìg protein/lane) from laminae of healthy horses (CON; n = 8), horses with OG1-lameness (n = 6) and horses with OG3-lameness (n = 5) showing expression of: **A**. integrin β4; **B**. β-catenin; **C**. vimentin; **D**. GSK 3β; **E**. serine-9-phospho GSK3β (pGSK 3β); **F**. E- cadherin; β actin was used as load control. The bands enclosed by the black box represent a common sample (from the OG1 group) that was run in all gels and used for normalizing experiment variation. The intensity of chemiluminescence was quantified and values were statistically analyzed shown in Fig. 1K, Fig. 2B, Fig. 3H, Fig. 5A, B and FigS4A respectively.
(TIF)

Figure S3 **Distribution of Wnt4 and FZD4 in healthy and laminitic equine digital laminae**: 10 µm sections of frozen laminae from: **A, D** - a representative (n = 3) healthy horse (CON), **B, E** - a representative (n = 4) horse with OG1-lameness (OG1) and **C, F** - a representative (n = 3) horse with OG3-lameness (OG3) were stained red with antibodies against Wnt4 (A, B, C) and FZD4 (D, E, F) (**panel F**–PEL = primary epidermal laminae). Nuclei are stained blue with DAPI. Images were taken with a 20× objective. Scale bars 50 µm.
(TIF)

Figure S4 **E-cadherin protein expression and integrin α6 gene expression:** **A** - Protein expression of E-cadherin relative to the protein expression of β actin. **B** - Gene expression of integrin α6 relative to the gene expression of GapDH. Horizontal lines indicate mean ± standard error of mean of n = 8 CON, n = 6 OG1, and n = 5 OG3 animals. * = p<0.05 as calculated by one-way ANOVA.
(TIF)
