# Supplementary figures and images for: Impact of Laminitis on the Canonical Wnt Signaling Pathway in Basal Epithelial Cells of the Equine Digital Laminae
Source: PLoS One. 2013 Feb 6;8(2):e56025. doi: 10.1371/journal.pone.0056025 (PMC3566061; doi:10.1371/journal.pone.0056025)

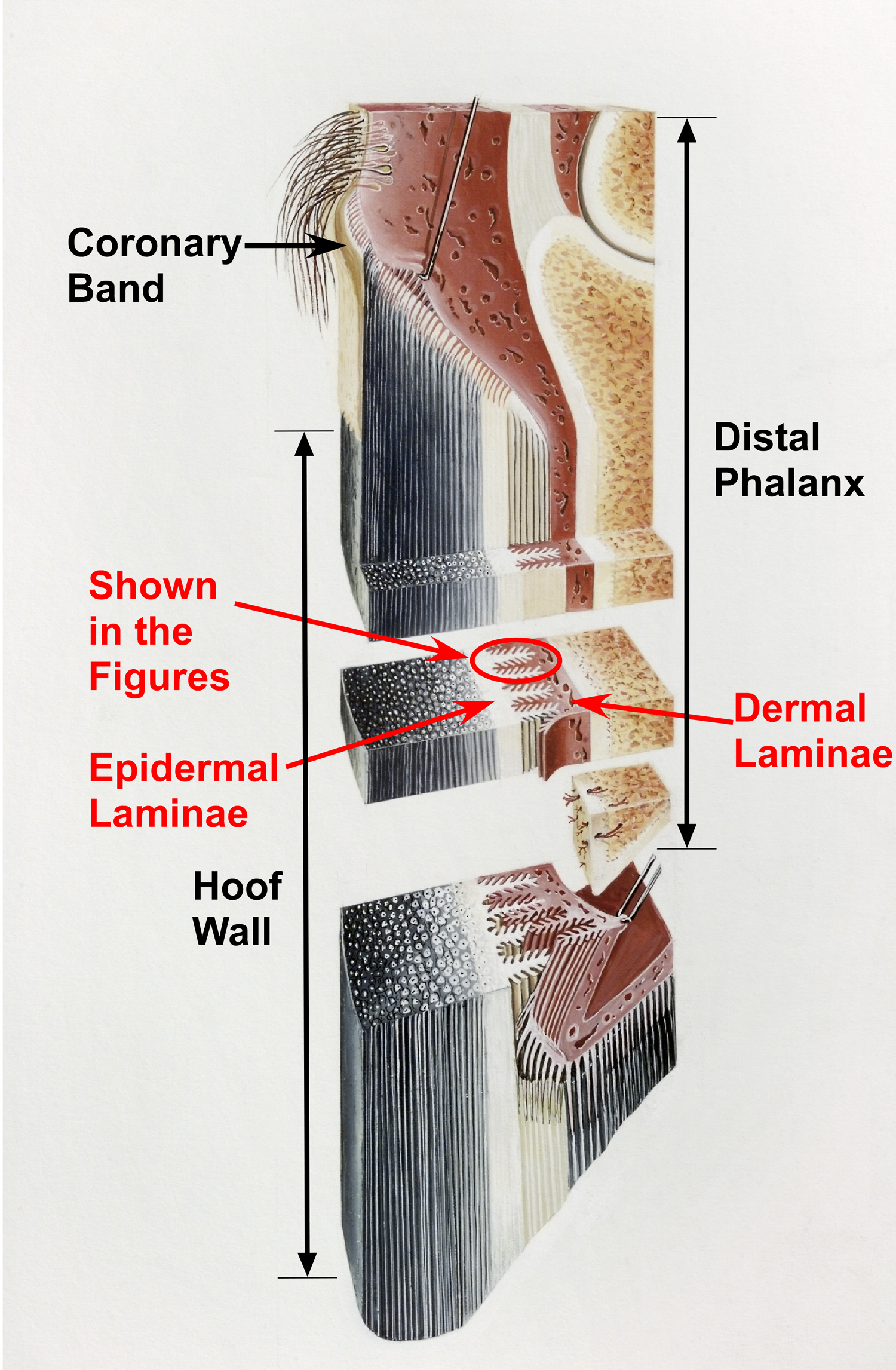

Supplement: Figure S1 — The anatomy of the hoof wall (Design: Chris Pollitt, Art. John McDougall): The Figure is reproduced by permission of Dr. C. Pollitt. Sections of laminae presented in the paper are from the mid dorsal front hoof laminae and the region is delineated by the oval shown in this figure. (TIF) [file pone.0056025.s001.tif]

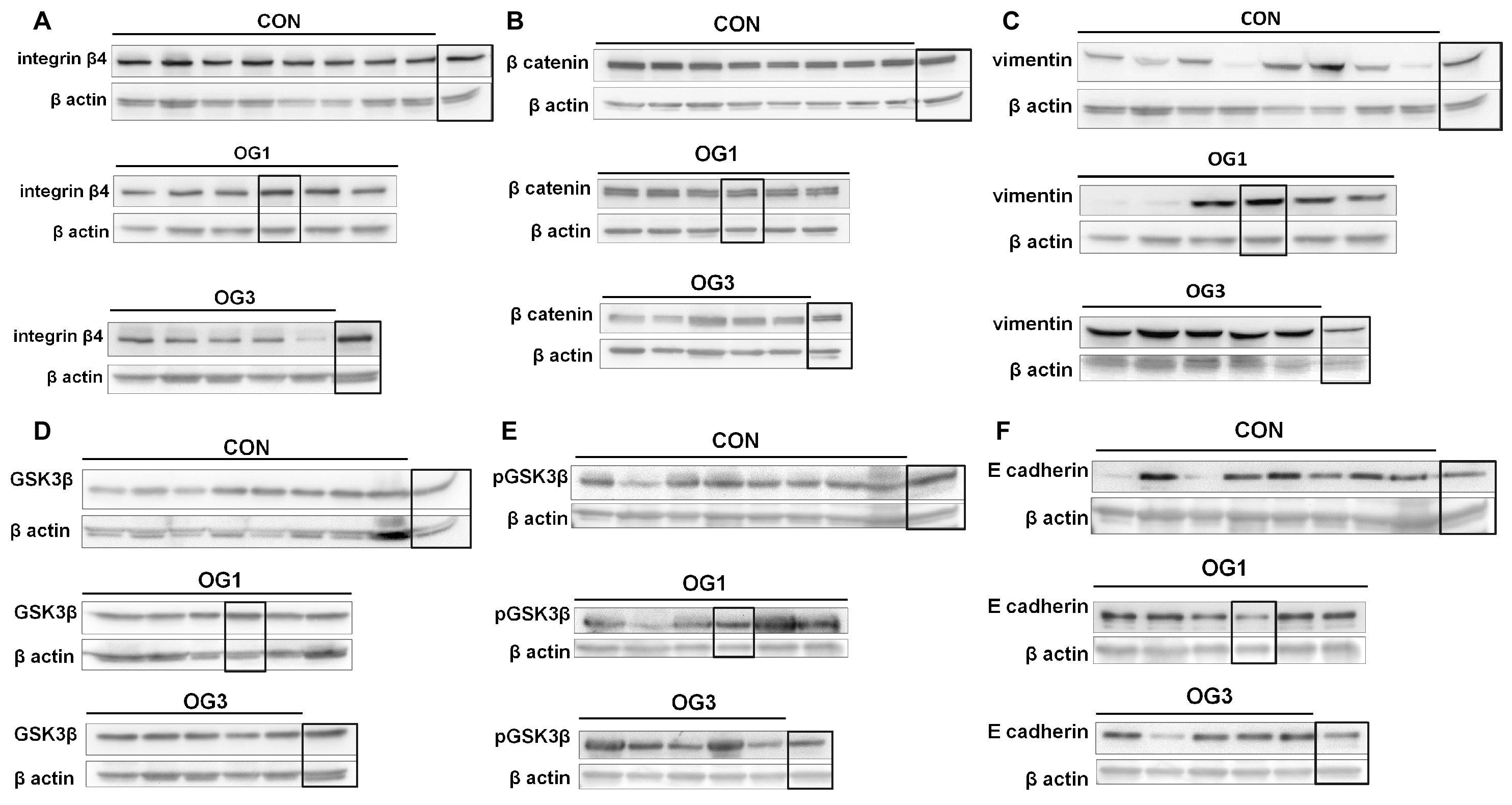

Supplement: Figure S2 — Protein expression in NP-40 extracts of healthy and laminitic equine digital laminae: Western blots of 0.5% NP-40 extracts (30 ìg protein/lane) from laminae of healthy horses (CON; n = 8), horses with OG1-lameness (n = 6) and horses with OG3-lameness (n = 5) showing expression of: A. integrin β4; B. β-catenin; C. vimentin; D. GSK 3β; E. serine-9-phospho GSK3β (pGSK 3β); F. E- cadherin; β actin was used as load control. The bands enclosed by the black box represent a common sample (from the OG1 group) that was run in all gels and used for normalizing experiment variation. The intensity of chemiluminescence was quantified and values were statistically analyzed shown in Fig. 1K, Fig. 2B, Fig. 3H, Fig. 5A, B and FigS4A respectively. (TIF) [file pone.0056025.s002.tif]

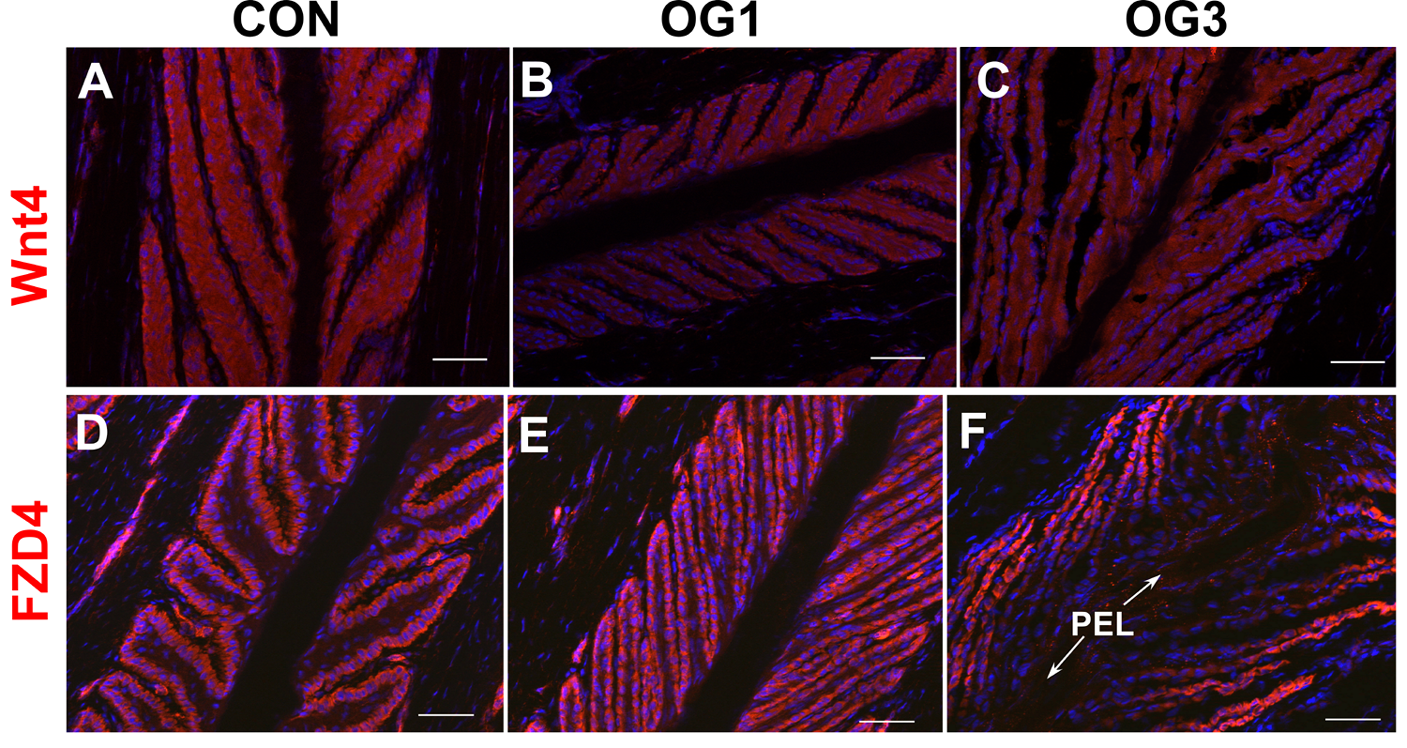

Supplement: Figure S3 — Distribution of Wnt4 and FZD4 in healthy and laminitic equine digital laminae: 10 µm sections of frozen laminae from: A, D - a representative (n = 3) healthy horse (CON), B, E - a representative (n = 4) horse with OG1-lameness (OG1) and C, F - a representative (n = 3) horse with OG3-lameness (OG3) were stained red with antibodies against Wnt4 (A, B, C) and FZD4 (D, E, F) (panel F–PEL = primary epidermal laminae). Nuclei are stained blue with DAPI. Images were taken with a 20× objective. Scale bars 50 µm. (TIF) [file pone.0056025.s003.tif]

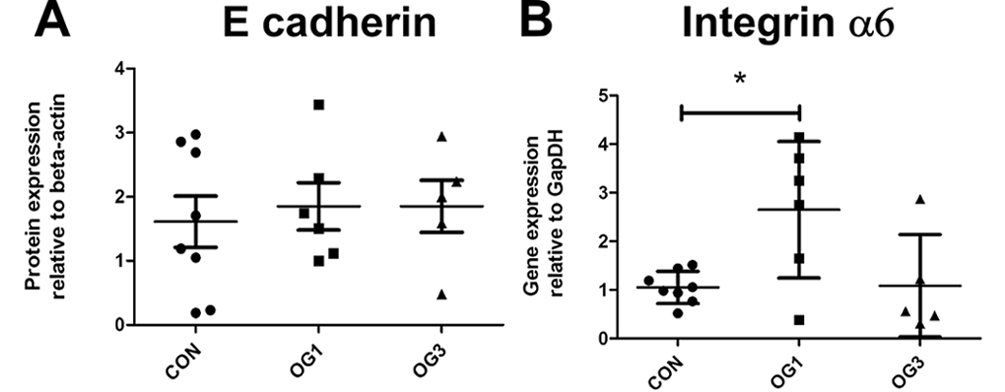

Supplement: Figure S4 — E-cadherin protein expression and integrin α6 gene expression: A - Protein expression of E-cadherin relative to the protein expression of β actin. B - Gene expression of integrin α6 relative to the gene expression of GapDH. Horizontal lines indicate mean ± standard error of mean of n = 8 CON, n = 6 OG1, and n = 5 OG3 animals. * = p<0.05 as calculated by one-way ANOVA. (TIF) [file pone.0056025.s004.tif]
